# Effects of matrix on plasma levels of EPA and DHA in dogs

**DOI:** 10.1017/jns.2017.30

**Published:** 2017-07-24

**Authors:** Kay Goffin, Marc van Maris, Ronald J. Corbee

**Affiliations:** 1 Department of Clinical Sciences of Companion Animals, Faculty of Veterinary Medicine, Utrecht University, Yalelaan 108, 3584 CM Utrecht, The Netherlands; 2 Ayanda Concordix AS, Brynsveien 11-13, N-0667 Oslo, Norway

**Keywords:** Fatty acids, Osteoarthritis, Micro-encapsulation, Emulsions, AUC0–24 h, AUC for 1 d, CCx, soft chew tablet, Cmax, maximum plasma concentration, EK, enriched kibbles, LFO, liquid fish oil, ME, metabolisable energy

## Abstract

EPA and DHA are often used in veterinary medicine due to their beneficial effects for several medical conditions such as osteoarthritis. EPA and DHA are administered to dogs through different matrices. The aim of the present study was to determine the effects on the plasma levels in dogs caused by various matrices for EPA and DHA administration. In this study, three different *n*-3 PUFA formulations were used: soft chew tablet (CCx); liquid fish oil (LFO); and enriched kibbles (EK). The formulations were administered single-dose and compared in a randomised, cross-over designed study with a 1-week wash-out period. Several variables were observed after the administration of these formulations in thirteen dogs: the NEFA plasma concentration, the AUC for 1 d (AUC0–24 h), and maximum plasma concentration for both EPA and DHA. All plasma fatty acid levels reached baseline levels within 72 h. CCx (median = 2·987) had a significantly lower AUC0–24 h for EPA compared with LFO (median = 5·647, *P* = 0·043) and EK (median = 5·119, *P* = 0·032) (*F*_2,22_ = 4·637, *P* = 0·021). CCx (median = 2·471) AUC0–24 h for DHA was significantly lower compared with LFO (median = 4·837, *Z* = −2·56, *P* = 0·011) and EK (median = 4·413, *Z* = −2·59, *P* = 0·01). EPA and DHA plasma levels were affected by matrix, as with the CCx, the AUC0–24 h of EPA and DHA were both lower compared with LFO and EK. The effect of matrix on bioavailability is important for product development as well as for clinical trials studying effects of EPA and DHA.

The benefits of *n*-3 PUFA supplements in human medicine have been extensively studied for (adjunctive) treatment of both non-inflammatory^(^^1^^,^^2^^)^ and inflammatory^(^^3^^)^ diseases. There is also evidence for beneficial effects of *n*-3 PUFA supplements in companion animals^(^^4^^)^. In particular, the *n*-3 PUFA EPA and DHA are thought to contribute to these beneficial effects, because conversion of α-linolenic acid to EPA and DHA is limited^(^^5^^)^. In dogs and cats, most evidence for beneficial effects of EPA and DHA is for its use in osteoarthritis. In these osteoarthritis studies, EPA and DHA were administered by the use of enriched kibbles (EK)^(^^6^^,^^7^^)^, or the use of liquid fish oil (LFO)^(^^8^^,^^9^^)^. As far as the authors know the effects of these matrices on the bioavailability of EPA and DHA have not been studied.

Bioavailability is defined by the Food and Drug Administration as ‘the rate and the extent to which the therapeutic moiety is absorbed and becomes available to the site of drug action’ or in a broader sense, it refers to the amount of a given substance that actually reaches systemic circulation or the site of physiological activity^(^^10^^)^. The overall bioavailability is determined by the fraction of compound which becomes accessible for intestinal absorption, the fraction of compound which can be transported across the small-intestinal epithelium, and the fraction of the absorbed compound which reaches the systemic circulation without being metabolised^(^^11^^)^. In a human study, bioequivalence of soft chew tablets (CCx) and LFO regarding EPA and DHA was demonstrated^(^^12^^)^. The benefit of CCx is that they are easier to administer EPA and DHA to children, as they dislike the taste of LFO and it is also difficult to administer them flavourless capsules. The same is true for cats^(^^9^^)^.

Bioavailability of nutraceuticals (i.e. products derived from food sources with extra health benefits in addition to the basic nutritional value found in foods) that contain highly lipophilic molecules is generally low. This is the result of a couple of physicochemical or physiological processes such as restricted release from the product matrix, low solubility in gastrointestinal fluids, low permeability across intestinal epithelial cells, and enzymic or chemical transformations within the gastrointestinal tract^(^^13^^)^. Apart from these factors, the bioavailability of *n*-3 PUFA depends on numerous factors such as the type of chemical bond, the concomitant intake of food and the presence of other components^(^^10^^)^. Some studies found that the bioavailability of *n*-3 PUFA is affected by the composition of the meal ingested together with fish oil capsules. High-fat meals (4·9 g fat per 418 kJ (100 kcal) metabolisable energy (ME)) demonstrated higher *n*-3 PUFA absorption (90 %) when compared with low-fat meals (0·9 g fat per 418 kJ (100 kcal) ME) (69 % absorption) in human subjects^(^^14^^,^^15^^)^. Furthermore, there are differences between different types of LFO. Several kinetic studies in human subjects demonstrated that NEFA have better bioavailability compared with TAG, or ethyl esters^(^^10^^,^^14^^,^^16^^,^^17^^)^.

In the case of EPA and DHA, there are several delivery systems such as LFO, powder, capsules, and micro- or nano-emulsions. Two studies in human subjects concluded that consumption of *n*-3 PUFA in an emulsified form is more effective compared with consumption of plain oil, and therefore enhances the bioavailability of *n*-3 PUFA^(^^18^^,^^19^^)^.

To provide more insight into the effects of matrices on the bioavailability of EPA and DHA in dogs, the aim of the present study was to determine the effects of three different matrices on the plasma levels of EPA and DHA in dogs.

## Materials and methods

### Animals

All study subjects were healthy Beagle dogs; six intact males and seven intact females. The number of dogs (*n* 13) was chosen after executing power analysis. This sample size was shown to be able to demonstrate a 33 % difference between the different treatments (Haug *et al.*^(^^12^^)^ demonstrated 40–45 % increase of plasma levels of EPA and DHA from CCx compared with LFO in human subjects) with a power of 0·80 and a level of significance of 0·05. Inclusion criteria for subject dogs was that the dogs must be at least 1 year old, with a good health status based on physical examination, complete blood count, blood biochemistry and urinalysis. Exclusion criteria were the presence of diarrhoea, and dogs that already consumed EPA and DHA supplements or EK within the last 14 d. The thirteen included dogs were between 2 and 8 years of age (median 4 years) and had a mean weight of 14·5 (sd 1·8) kg at the start of the study. The dogs were reweighed at the end of the study. The dogs were selected from the University's colony, and were housed at the University clinic for companion animals with indoor and outdoor access. They were fed a standard diet (Hill's Science Plan Canine Adult Advanced Fitness Medium Lamb and Rice) for at least 6 months prior to the study, at approximately 3347 kJ (800 kcal) ME per d, which was determined by 1·54 × (average body weight of the dogs in kg)^0·75^ × 293 kJ (70 kcal) ME; one meal per d.

### Study design

In this study, three different *n*-3 PUFA matrices (CCx, LFO and EK) were compared in a randomised, cross-over designed study. The dogs were randomised by computer randomisation into six groups, following a different order (i.e. group 1 (*n* 3) first CCx, followed by LFO, and finally EK; group 2 (*n* 2) CCx, EK, LFO; group 3 (*n* 2) LFO, EK, CCx; group 4 (*n* 2) LFO, CCx, EK; group 5 (*n* 2) EK, CCx, LFO; group 6 (*n* 3) EK, LFO, CCx). After the administration of each of the three different matrices in thirteen dogs the NEFA plasma concentration at different time points, the AUC for 1 d (AUC0–24 h) and the maximum plasma concentration (Cmax) were determined for both EPA and DHA. After initial measurements (*T* = 0), the dogs received a single dose of the supplement containing 1500 mg EPA + DHA (900 mg EPA + 600 mg DHA), or in a portion of 3347 kJ (800 kcal) ME for EK. The amounts of EPA and DHA were determined by the EK, as we could not modify the composition of this diet. All the dogs were fed one meal of 3347 kJ (800 kcal) ME per d excluding the energy coming from the supplements (i.e. LFO and CCx). The dogs were kept on the standard diet throughout the rest of the study. After the single dose of the supplement (or after a single meal of EK), blood samples were taken and analysed for the NEFA of EPA and DHA at different time points (baseline, 1, 2, 3, 6, 9, 12, 24, 72 h, 1 week). This was repeated for each of the three matrices, with a 1 week wash-out period between the different matrices. The length of the washout period was determined based on preliminary unpublished findings that EPA and DHA plasma levels reached baseline within 72 h after single-dose administration. This study was approved by the ethical committee as required under Dutch legislation and registered under number DEC 2014.III.03.018 (date of approval: 28-02-2014).

### Diets and supplements

The composition of the diets and supplements can be found in Appendices 1 and 2. The amount of *n*-3 PUFA, EPA and DHA in the diets and supplements are shown in Table 1. The amount of *n*-3 PUFA, EPA and DHA of the CCx, LFO and EK were determined by the GC Rieber Laboratory (Kristiansund, Norway) prior to the start of the study.

**Table 1.** Amount of *n*-3 PUFA in foods and supplements*

Fatty acid	CCx (mg)	LFO (mg)	EK (mg)	Standard diet (mg)
EPA	900	900	856	6·4
DHA	600	600	548	4
EPA + DHA	1500	1500	1404	10·4
Total *n*-3	1800	1800	7272	1024
Product weight (g)	7·5	3	222	213

CCx, soft chew tablet; LFO, liquid fish oil; EK, enriched kibbles.

* Amount of fatty acids in the treatments and standard diet per serving (dietary intake of 3347 kJ (800 kcal) metabolisable energy per d).

The diets and LFO were purchased from the manufacturer and throughout the study only bags/bottles from one specific batch was fed to the dogs. The CCx were specifically manufactured for this study in one batch. The LFO and CCx were stored at 4°C and the diets were stored at room temperature in the original package (with zip seal) without direct sunlight.

### Blood collection

Blood samples were obtained by jugular venepuncture. Samples of 1 ml were collected in EDTA-coated tubes (1 ml) which were immediately centrifuged at 3500 rpm for 8 min at 4°C to separate blood plasma from other blood components. The plasma was stored at −20°C until fatty acid analysis was performed.

### Fatty acid analysis

Cholesteryl esters in plasma were isolated by a modified ‘Bligh & Dyer’ extraction according to Retra *et al.*^(^^20^^)^, followed by a solid-phase extraction method according to Hamilton & Comai^(^^21^^)^. Thereafter, the cholesteryl esters were saponified according to the modified method described by Kates^(^^22^^)^, where petroleum ether was replaced by hexane. PUFA analysis was performed by HPLC/MS according to Retra *et al.*^(^^20^^)^, in which the Synergi 4 µm MAX-RP 18 Å column was replaced by a Kinetex 2·6 µm C18 100 Å column (150 × 3 mm; Phenomenex). Internal standards were used for comparison.

### Statistics

Statistical analysis was performed using IBM^®^ SPSS Statistics for Windows, version 23.0. Repeated-measures ANOVA was performed for normally distributed data (baseline measures and Cmax for EPA and DHA, plasma concentrations of DHA at different time points). For non-normally distributed data (AUC0–24 h for EPA and DHA, plasma concentrations of EPA at different time points), the Friedman test was performed. Analysis included comparison of plasma concentration intervals, AUC0–24 h and Cmax for both EPA and DHA. The level of significance was set at *P* < 0·05. The data are presented as the test statistic (with df), followed by the *P* value.

## Results

The dogs’ body weights did not change during the study. One dog did not eat the food when the LFO was poured on top of it and mixed. This dog was thus deleted in the LFO results. Three other dogs ate their food partially when LFO was added; two dogs only partially ate the EK. The results for these particular dog–formulation combinations were left out of the analysis, leaving *n* 9 for LFO, *n* 11 for EK and *n* 13 for CCx for analysis. With all formulations, plasma levels of EPA and DHA reached baseline levels 72 h post-administration.

**Fig. 1.**
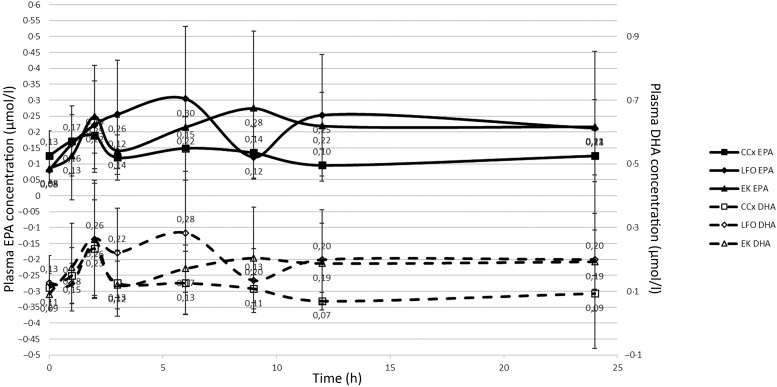
Plasma levels of EPA and DHA during the first 24 h after a single-dose administration for three different matrices (i.e. soft gel tablet (CCx, *n* 13 dogs), liquid fish oil (LFO, *n* 9) and enriched kibbles (EK, *n* 11)) expressed in μmol/l. Values are means, with standard deviations represented by vertical bars.

The plasma levels of EPA and DHA during the first 24 h post-administration are demonstrated in Fig. 1.

The baseline plasma measures (*T* = 0, EPA plasma levels before the start of the study) for EPA (*F*_2,22_ = 1·415, *P* > 0·05) did not differ between groups. Plasma concentrations of EPA at different time points after supplementation did not differ between groups (*F*_1·4,15·1_ = 3·217, *P* = 0·083). CCx (median = 2·987) had a significantly lower AUC0–24 h compared with LFO (median = 5·647, *P* = 0·043) and EK (median = 5·119, *P* = 0·032) (*F*_2,22_ = 4·637, *P* = 0·021). Cmax did not differ between groups (*F*_2,22_ = 2·026, *P* = 0·156).

For DHA, *T* = 0 (DHA plasma levels before the start of the study) did not differ between groups (*F*_2,22_ = 1·303, *P* > 0·05). Plasma concentrations at different time points after supplementation did not differ between groups (*F*_2,22_ = 3·3, *P* = 0·056). CCx (median = 2·471) AUC0–24 h was significantly lower compared with LFO (median = 4·837, *Z* = −2·56, *P* = 0·011) and EK (median = 4·413, *Z* = −2·59, *P* = 0·010). Cmax did not differ between groups (*F*_2,22_ = 3·306, *P* = 0·056).

## Discussion

In this study, three matrices of fish oil supplements were compared, and the results demonstrated that there was no difference in EPA and DHA plasma concentrations at the different time points. Also, there was no significant difference in the Cmax of both EPA and DHA. This could be explained by the low number of dogs, especially because in the LFO group four dogs were left out of the analysis (underpowered for Cmax). However, AUC0–24 h of both EPA and DHA in the CCx condition were significantly lower compared with those for the LFO and EK conditions. This outcome disagrees with the results of the study by Haug *et al.* in human subjects^(^^12^^)^. In this study the CCx administration resulted in higher plasma levels of EPA and DHA compared with LFO^(^^12^^)^. The lower AUC0–24 h suggests a lower bioavailability of EPA and DHA when administered by CCx in dogs. However, as Cmax did not differ, it may also suggest that EPA and DHA reach their target tissues earlier when administered by CCx^(^^23^^)^. As EPA and DHA and their metabolites should be available at the site of action (e.g. in the synovial fluid), it would be worthwhile to measure this. For the present study, this was considered too invasive a procedure.

The lower bioavailability could not be explained by refusal of CCx. All dogs used in this study voluntarily consumed CCx, while LFO and EK were refused or partially taken by some dogs. CCx may therefore increase compliance when EPA and DHA should be administered on a daily basis by pet owners^(^^24^^,^^25^^)^.

Nutraceuticals often do not show typical dose–response curves as is seen with drugs^(^^26^^)^. The same is demonstrated in the present study. Interestingly, all three matrices demonstrate a secondary peak in both EPA and DHA. This is possibly due to a first peak reflecting dietary intake and a secondary one demonstrating newly formed EPA and DHA from precursors present in the diets and supplements (e.g. α-linolenic acid and eicosatetraenoic acid) and/or by chemical transformations within the gastrointestinal tract (e.g. re-esterification)^(^^13^^)^.

The effects of formulation or matrix on bioavailability are factors that affect the dissolution of the supplement. Factors related to excipients or inactive ingredients might affect supplement stability and absorption. For nutraceuticals, much more research is necessary to define the effect of formulation on bioavailability^(^^27^^)^. As far as the authors know, studies about enhancement of bioavailability of *n*-3 PUFA by changing matrix or formulation as well as effects of a single dose of EPA and DHA have not been studied in companion animals before. In dogs, increased plasma levels of EPA and DHA were demonstrated after a prolonged period of supplementation (e.g. more than 14–21 d)^(^^5^^,^^8^^)^ (about 6-fold increase for EPA and 2-fold increase for DHA compared with about 2-fold increase for both EPA and DHA in our study after 24 h).

Single-dose testing may not be the ideal way to test supplements’ bioavailability features. It is recommended, knowing that EPA and DHA are incorporated into tissues (e.g. erythrocyte membranes), that future studies of bioavailability of fish oil supplements in dogs should have a study design with a longer period of administration^(^^10^^,^^23^^,^^28^^)^. The concentration of EPA and DHA in erythrocyte membranes correlated well with the concentration of EPA and DHA in target tissues in rats^(^^23^^)^. Determination of the concentration of EPA and DHA in erythrocyte membranes or target tissues may therefore be a more reliable method to determine their bioavailability.

### Conclusion

EPA and DHA plasma levels were affected by the matrix, as the AUC0–24 h of EPA and DHA were both lower for CCx compared with LFO and EK. The effect of matrix on bioavailability is important for product development as well as for clinical trials studying the effects of EPA and DHA.

## Supplementary material

For supplementary material accompanying this paper visit http://dx.doi.org/10.1017/jns.2017.30. click here to view supplementary material
