# Supplementary material for: Effects of matrix on plasma levels of EPA and DHA in dogs
Source: J Nutr Sci. 2017 Jul 24;6:e37. doi: 10.1017/jns.2017.30 (PMC5672307; doi:10.1017/jns.2017.30)
Supplement: Supplementary file 1 [file S2048679017000301sup001.docx]

Appendix A

The ingredients of ConCordix© (soft chew tablet), Hill’s Prescription Diet J/D Canine Dry (enriched kibble), and VivoMega 3322 TG60 (liquid fish oil), and Hill’s Science Plan Canine Adult Advanced Fitness Medium Lamb and Rice (standard diet) were:

- Soft chew tablet; Fish oil TG3322, purified water, Erythritol E968, Maltodextrin, Bovine Gelatin E441, Dextrose, Sucrose, Calanus Hydrolysate, Caramel E150C, Vegetable oil.

- Enriched kibble; Whole Grain Corn, Chicken By-Product Meal, Flaxseed, Soybean Mill Run, Brewers Rice, Soybean Meal, Pork Fat, Chicken Liver Flavor, Powdered Cellulose, Fish Oil, Lactic Acid, Potassium Chloride, L-Lysine, Calcium Carbonate, Choline Chloride, Iodized Salt, DL-Methionine, vitamins (Vitamin E Supplement, L-Ascorbyl-2-Polyphosphate (source of vitamin C), Niacin Supplement, Thiamine Mononitrate, Vitamin A Supplement, Calcium Pantothenate, Biotin, Vitamin B12 Supplement, Pyridoxine Hydrochloride, Riboflavin Supplement, Folic Acid, Vitamin D3 Supplement), L-Threonine, Taurine, Soy Lecithin, Glucosamine Hydrochloride, minerals (Ferrous Sulfate, Zinc Oxide, Copper Sulfate, Manganous Oxide, Calcium Iodate, Sodium Selenite), L-Tryptophan, L-Carnitine, Mixed Tocopherols for freshness, Chondroitin Sulfate, Phosphoric Acid, Beta-Carotene, Natural Flavors.

- Liquid fish oil; High EPA concentrate on triglyceride (TG) basis made from fish oil. The specification is a min 60% TG product with 3.0 – 4.0 mg/g of mixed tocopherols added.

- Standard diet; Lamb Meal, Brown Rice, Brewers Rice, Whole Grain Wheat, Whole Grain Sorghum, Corn Gluten Meal, Pork Fat, Cracked Pearled Barley, Chicken Liver Flavor, Soybean Oil, Flaxseed, Dried Beet Pulp, Lactic Acid, Potassium Chloride, Iodized Salt, L-Lysine, Choline Chloride, vitamins (Vitamin E Supplement, L-Ascorbyl-2-Polyphosphate (source of Vitamin C), Niacin Supplement, Thiamine Mononitrate, Vitamin A Supplement, Calcium Pantothenate, Vitamin B12 Supplement, Pyridoxine Hydrochloride, Riboflavin Supplement, Biotin, Folic Acid, Vitamin D3 Supplement), Taurine, minerals (Ferrous Sulfate, Zinc Oxide, Copper Sulfate, Manganous Oxide, Calcium Iodate, Sodium Selenite), L-Tryptophan, Mixed Tocopherols for freshness, L-Threonine, Natural Flavors, Beta-Carotene.

Composition of the diets and supplements

| Per 100 kcal ME | EK | Standard diet | CCx | LFO |
| --- | --- | --- | --- | --- |
|  |  |  |  |  |
| Protein | 5.1 g | 5.8 g | 0.4 g | 0.0 g |
| Fat | 4.1 g | 4.0 g | 6.5 g | 11.1 g |
| Carbohydrates (NFE) | 13.5 g | 13.1 g | 6.5 g | 0.0 g |
| Crude fiber | 1.5 g | 0.5 g | 0.9 g | 0.0 g |
| Moisture | 2.2 g | 2.1 g | 2.8 g | 0.0 g |
| Omega-3 fatty acids | 909 mg | 128 mg | 3,871 mg | 6,327 mg |
| Omega-6 fatty acids | 616 mg | 854 mg | N/A | N/A |
| EPA | 107 mg | 0.8 mg | 1,935 mg | 3,219 mg |
| DHA | 72 mg | 0.5 mg | 1,290 mg | 2,109 mg |

Composition per 100 kcal Metabolizable Energy (ME) of the enriched kibble (EK), the standard diet, the soft chew tablet (CCx), and the liquid fish oil (LFO). Carbohydrates are demonstrated as Nitrogen Free Extract (NFE). Some data are not available (N/A).
